# Effect of Storage on Acrylamide and 5-hydroxymethylfurfural Contents in Selected Processed Plant Products with Long Shelf-life

**DOI:** 10.1007/s11130-015-0523-4

**Published:** 2016-01-14

**Authors:** Joanna Michalak, Elżbieta Gujska, Marta Czarnowska, Joanna Klepacka, Fabian Nowak

**Affiliations:** Food Science Department, University of Warmia and Mazury, ul. Heweliusza 6, 10-957 Olsztyn, Poland

**Keywords:** Acrylamide, 5-hydroxymethylfurfural, Storage conditions, Processed plant products

## Abstract

This study investigated the effects of storage and temperature duration on the stability of acrylamide (AA) and 5-hydroxymethylfurfural (HMF) in selected foods with long shelf-life. Products were analysed fresh and stored at temperatures of 4 and 25 °C after 6 and 12 months (with the exception of soft bread samples, which were analysed after 15 and 30 days). The AA and HMF contents were determined with RP-HPLC coupled to a diode array detector (DAD). AA and HMF were not stable in many processed plant products with a long shelf-life. The highest AA reduction and the largest increase in HMF content were observed in the samples stored at a higher temperature (25 °C) for 12 months. It was found that an initial water activity of 0.4 is favourable to HMF formation and that AA reduction may be considerably greater in stored products with a low initial water activity. The kind of product and its composition may also have a significant impact on acrylamide content in stored food. In the final period of storage at 25 °C, acrylamide content in 100 % cocoa powder, instant baby foods, 20 % cocoa powder and instant coffee was 51, 39, 35 and 33 % lower than in products before storage, respectively. It was observed that a large quantity of ε-NH_2_ and SH groups of amino acids in some products can be assumed as the reason for the significant AA degradation.

## Introduction

The Maillard reaction is considered to be one of the most important chemical reactions taking place during food processing. Some Maillard reaction products may have high toxicological potential [1, 2]. Acrylamide (AA) has been added to the list of food-borne toxicants since 2002. It is found in concentrations up to a few mg/kg in a wide range of foods, mainly carbohydrate-rich foods subjected to high thermal processing [1, 3, 4]. The acrylamide content in foods is defined as the net amount of acrylamide, *i.e.*, what remains after formation and degradation. In general, it has been found that prolonged heating and storage can also cause AA degradation. Opinions, however, differ and there is little data available on this subject. The type of reaction responsible for the degradation is also still unclear and it is believed that these reactions may be heterogeneous for different products [5–7]. Another compound formed during the Maillard reactions is 5-hydroxymethylfurfural (HMF). HMF concentrations in foods vary widely, sometimes exceeding 1 g/kg in certain dried fruits and caramel products. However, the most important contributors to dietary HMF intake are bread and coffee [8, 9]. Many studies have shown that it can be toxic, mutagenic and carcinogenic to the human body. However, other researchers have found that HMF and its derivatives may be an anti-carcinogenic factor [8, 10, 11]. To date, there are no available mitigation strategies specifically addressed to reduce HMF content in foods [12].

Plant foods are important in human and animal nutrition [13]. However, AA and HMF are present in high quantities in many plant-based processed foods [8]. Plant-based processed foods include snacks, pasta and breakfast cereals, as well as breads, coffee, cocoa and baby food. Since some processed plant foods are stored for long periods of time, it is important to investigate AA and HMF changes during storage. In several previous studies, the effect of some storage variables on AA and HMF formation was investigated and the effects were individually tested but not in combination with each other. Therefore, from these studies, it has not been possible to understand the most optimum conditions leading to minimizing HMF formation and maximizing AA degradation in products during storage. Thus, in this study, the effect of storage on acrylamide and 5-hydroxymethylfurfural contents in different food matrixes with long shelf-life was investigated. The influence of initial water activity of products upon acrylamide and HMF kinetics was also examined.

## Materials and Methods

### Samples

The test material included 90 samples of commercially-produced plant-based foods: a) cereal-based foods for infants such as: (10) jarred baby foods (ready-to-eat), (10) instant baby foods (in powder products) and (10) biscuits for infants. The major ingredients of these foods were cereals, milk powder and various sugars, in some cases follow-on formula and dried fruit and/or natural fruit powder; b) (10) cacao (100 % cocoa powder), (10) cacao (cocoa-containing beverages powder: sugars and 20 % cocoa powder); c) (10) instant (soluble) coffee, (10) coffee substitutes containing different proportions of coffee and sugars, chicory and other substitutes such as rye, barley, malt; d) (10) crisp bread (wheat, rye, multigrain bread) and (10) soft bread (also wheat, rye, multi-grain bread). The soft bread with a prolonged shelf-life was packed in modified atmosphere.

Five units of each product type were purchased from local supermarkets. The production date of these products was not longer than seven days. One was analysed immediately after buying (zero time). The first two units were stored at 4 °C (fridge) and the second two units were stored at 25 °C (thermostat). The samples were stored in commercial package in the dark and were analysed after 6 and 12 months of storage. The soft bread samples, due to their relatively short shelf-life, were analysed immediately after the production (zero time) and after 15 and 30 days. The instant samples (instant baby foods, cacao, coffee) were analysed as bought after being thoroughly stirred. Other products were crushed or/and homogenized in a Masterchef 8000 FP664 Moulinex multi-function food processor (Grupe SEB Poland Sp. z o.o., Warsaw, Poland). All samples were analysed before the expiry date. Sample preparation and analytical determinations were carried out in triplicate.

### Reagents and Chemicals

The acrylamide standard (99.8 %, catalogue No. 23701), 5-hydroxymethylfurfural (≥98.0 %, catalogue No. 53407) and all chemicals of HPLC analytical grade were obtained from Sigma–Aldrich (St. Louis, MO, USA) and Merck (Darmstadt, Germany).

### Determination of Water Activity (a_w_)

Water activity (a_w_) was determined using a resistive electrolytic humidity measuring system (Novasina LabMaster AW, Novatron Scientific Ltd., Horsham, England).

### Determination of Acrylamide

The AA content in all food products was determined with the method developed by Michalak et al. [14] using the ion-pair RP–HPLC (Shimadzu LC–10A, Kyoto, Japan). The quality parameters were as follows: detection limit 10 μg/kg, the linear range: 10.0–40,000 μg/kg with satisfactory linearity (*R*^*2*^ = 0.9997) and 106 % recovery.

### Determination of 5-hydroxymethylfurfural

5-hydroxymethylfurfural was determined by the procedure of Ferrer et al. [15] and Chavez-Servin et al. [16] using the RP–HPLC (Agilent 1200, Santa Clara, CA, USA). The quality parameters of the method were: detection limit 0.01 mg/kg, the linear range of 0.03–250 mg/kg with satisfactory linearity (*R*^*2*^ = 0.9989), recovery - 101 %.

### Statistical Analysis

The data were analysed using the Statistica 9.0 software package (StatSoft, Poland). Significant differences were calculated according to Duncan’s multiple range test with a 5 % level considered statistically significant. The results were submitted to a bi-factorial (time and temperature) analysis of variance (ANOVA). The mean values were compared using the least significant difference test (LSD) at the 5 % level. Correlations among HMF, AA and water activity were determined by Pearson’s correlation analysis at the *p* < 0.05 confidence level.

## Results and Discussion

During storage, no change was detected in the HMF content of the jarred baby foods analysed (Table 1). According to Ramírez-Jiménez et al. [17], storing products with high water activity at relatively low temperature (25 °C) does not favour HMF formation. As can be seen in Table 1, acrylamide contents also did not significantly differ after 6 and 12 months of storage at 4 °C. At a higher temperature (25 °C), acrylamide loss during storage was observed. The initial acrylamide content of jarred baby foods decreased by 9 % and 15 % after 6 and 12 months of storage, respectively. It was found that storing jarred baby foods with high water activity (0.987) at a relatively low temperature (4 and 25 °C) did not favour HMF formation or a high reduction of AA during the 12-month study period.

**Table 1:** Changes of HMF (mg/kg), AA (μg/kg) and water activity (a_w_) in cereal-based foods for infants during storage

Type of product	Storage time (months)	Parameter^1^	Temperature	
			4 °C	25 °C
Jarred baby foods (ready-to-eat) (*n* = 10)	0	HMF AA a_w_	25.1 ± 1.1^Aa^ 15.1 ± 1.2^Aa^ 0.987 ± 0.005^Aa^	25.1 ± 1.1^Aa^ 15.1 ± 1.2^Aa^ 0.987 ± 0.005^Aa^
	6	HMF AA a_w_	25.3 ± 1.1^Aa^ 14.5 ± 1.0^Aa^ 0.985 ± 0.005^Aa^	25.6 ± 1.2^Aa^ 13.7 ± 0.9^Aab^ 0.983 ± 0.005^Aa^
	12	HMF AA a_w_	25.7 ± 1.4^Aa^ 14.3 ± 0.9^Aa^ 0.983 ± 0.005^Aa^	26.3 ± 1.5^Aa^ 12.9 ± 0.4^Bb^ 0.981 ± 0.005^Aa^
Instant baby foods (in powder products) (*n* = 10)	0	HMF AA a_w_	52.1 ± 13.9^Aa^ 34.7 ± 5.8^Aa^ 0.166 ± 0.028^Aa^	52.1 ± 13.9^Aa^ 34.7 ± 5.8^Aa^ 0.166 ± 0.028^Aa^
	6	HMF AA a_w_	55.3 ± 10.0^Aa^ 28.7 ± 2.1^Aab^ 0.161 ± 0.025^Aa^	56.6 ± 9.1^Aa^ 26.3 ± 2.2^Ab^ 0.160 ± 0.023^Aa^
	12	HMF AA a_w_	56.7 ± 11.4^Aa^ 25.3 ± 1.1^Ab^ 0.159 ± 0.035^Aa^	60.0 ± 10.5^Aa^ 21.1 ± 0.9^Bc^ 0.159 ± 0.033^Aa^
Biscuits for infants (*n* = 10)	0	HMF AA a_w_	87.2 ± 3.2^Ab^ 73.2 ± 5.8^Aa^ 0.366 ± 0.040^Aa^	87.2 ± 3.2^Ac^ 73.2 ± 5.8^Aa^ 0.366 ± 0.040^Aa^
	6	HMF AA a_w_	90.9 ± 4.1^Bb^ 68.0 ± 2.1^Aa^ 0.344 ± 0.035^Aa^	101.0 ± 5.9^Ab^ 64.1 ± 1.9^Ab^ 0.341 ± 0.033^Aa^
	12	HMF AA a_w_	96.7 ± 4.5^Ba^ 61.6 ± 3.8^Ab^ 0.339 ± 0.068^Aa^	113.0 ± 5.0^Aa^ 55.7 ± 3.1^Bc^ 0.331 ± 0.063^Aa^

^1^Mean values ± standard deviation (*n* = 10, *n* = the number of the same kind products from different producers)

^A–B^in a row, different letters indicate significant differences (*p* < 0.05)

^a–c^in a column within the same parameter, different letters indicate significant differences (*p* < 0.05) for the same type of products; LSD interaction time–temperature factor of HMF, AA and a_w_ are 1.14, 0.95, 1.50, respectively for jarred baby foods; LSD interaction time–temperature factor of HMF, AA and a_w_ are 1.37, 0.69, 1.45, respectively for instant baby foods; LSD interaction time–temperature factor of HMF, AA and a_w_ are 1.17, 0.65, 1.55, respectively for biscuits

At the same time, another baby product with very low water activity (0.166) was examined (Table 1). In this case, the storage of instant baby foods in powder also did not significantly affect HMF formation. It is believed that in these products, with very high and low water activity and stored at 4 °C and 25 °C, HMF formation is limited. The AA content in the examined instant baby foods was 34.7 μg/kg and during storage for 12 months at a temperature of 4 °C and 25 °C, it significantly decreased to 25.3 μg/kg and 21.1 μg/kg, respectively (Table 1). AA losses were greater at the higher storage temperature, reaching 39 % after 12 months. It was observed that a higher temperature and longer storage time had a greater impact on the degree of acrylamide reduction in the products.

In the case of stored biscuits for infants with an initial water activity of 0.366, HMF formation was observed (Table 1). The results showed that intermediate water activity, *i.e.*, about 0.4, can be more favourable to HMF formation than lower and higher a_w_ values. Acrylamide in biscuits was not a stable compound. AA content in biscuits stored at 25 °C for 12 months was about 24 % lower than before storage. The effect of storage on AA content in baby foods has not been intensely studied, but some studies have found no common pattern in the degree of AA reduction. The reduction of AA in different biscuits has been found to range from no decrease to a decrease of 20 % [7].

The instant cacao (100 % cocoa) contained, on average, 2.0 mg HMF/kg (Table 2). In other cacao products (cocoa-containing beverages powder: sugars and 20 % of cocoa), the mean HMF concentration was 153 mg/kg. In instant cacao samples, HMF content did not significantly differ after 6 and 12 months of storage at 4 °C and 25 °C (Table 2). The highest HMF content was found for the sweetened cacao products after 12 months of storage, both at 4 °C and 25 °C, (a 28 % and 46 % increase, respectively). HMF presence in foods is usually the result of the Maillard reaction or caramelization processes. Most likely, in the case of instant cacao (100 % cocoa), HMF was formed during the roasting process of the cocoa beans, whereas in sweetened cacao, HMF could be formed by both reactions. According to Abraham et al. [18], HMF concentration in cocoa-containing beverages powder and sugars can reach 503.8 mg/kg.

**Table 2:** Changes of HMF (mg/kg), AA (μg/kg) and water activity (a_w_) in cocoa products during storage

Type of product	Time (months)	Parameter^1^	Temperature	
			4 °C	25 °C
Cacao (100 % cocoa powder) (*n* = 10)	0	HMF AA a_w_	2.0 ± 0.5^Aa^ 347.0 ± 42.0^Aa^ 0.155 ± 0.015^Aa^	2.0 ± 0.5^Aa^ 347.0 ± 42.0^Aa^ 0.155 ± 0.015^Aa^
	6	HMF AA a_w_	2.1 ± 0.3^Aa^ 268.0 ± 34.0^Ab^ 0.154 ± 0.017^Aa^	2.1 ± 0.2^Aa^ 225.0 ± 34.0^Bb^ 0.154 ± 0.014^Aa^
	12	HMF AA a_w_	2.1 ± 0.2^Aa^ 202.0 ± 32.0^Ac^ 0.153 ± 0.018^Aa^	2.3 ± 0.2^Aa^ 169.0 ± 31.0^Bc^ 0.152 ± 0.013^Aa^
Cacao (cocoa-containing beverages powder: sugars and 20 % cocoa powder) (*n* = 10)	0	HMF AA a_w_	153.0 ± 8.0^Ac^ 248.0 ± 29.0^Aa^ 0.324 ± 0.083^Aa^	153.0 ± 8.0^Ac^ 248.0 ± 29.0^Aa^ 0.324 ± 0.083^Aa^
	6	HMF AA a_w_	175.0 ± 7.0^Bb^ 228.0 ± 12.0^Aa^ 0.321 ± 0.075^Aa^	192.0 ± 9.0^Ab^ 215.0 ± 10.0^Bb^ 0.320 ± 0.083^Aa^
	12	HMF AA a_w_	196.0 ± 9.0^Ba^ 191.0 ± 19.0^Ab^ 0.319 ± 0.078^Aa^	224.0 ± 8.0^Aa^ 162.0 ± 15.0^Bc^ 0.319 ± 0.072^Aa^

^1^Mean values ± standard deviation (*n* = 10, *n* = the number of the same kind products from different producers)

^A–B^in a row, different letters indicate significant differences (*p* < 0.05)

^a–c^: in a column within the same parameter, different letters indicate significant differences (*p* < 0.05) for the same type of products; LSD interaction time–temperature factor of HMF, AA and a_w_ are 0.88, 0.35, 1.50, respectively for cocoa powder; LSD interaction time–temperature factor of HMF, AA and a_w_ are 0.73, 0.42, 1.45, respectively for cocoa 20 % powder

During the roasting process of cacao beans, acrylamide is also formed. For instant cacao (100 % cocoa), the average acrylamide concentration was 347 μg/kg, while in sweetened cacao it was 248 μg/kg. After storage at 4 °C and 25 °C for 12 months, AA concentrations in instant cacao significantly dropped to 202 μg/kg (42 % decrease) and 169 μg/kg (51 % decrease), respectively; in sweetened cacao to 191 μg/kg (23 % decrease) and 162 μg/kg (35 % decrease), respectively (Table 2). This finding was similar to the observation made by Hoenicke and Gatermann [6], who showed that the acrylamide concentrations in cacao powder stored at 10–12 °C for 6 months dropped significantly from 265 μg/kg to 180 μg/kg (a 32 % decrease).

Table 3 shows that the amount of HMF was significantly higher in the coffee substitutes (397 mg/kg) than in the instant coffee (120 mg/kg). Husøy et al. [19] and Del Campo et al. [20] reported a great variation of HMF levels within instant coffee food products: from 97 to 6180 mg/kg. As shown in Table 3, significant formation of HMF during storage at 25 °C for 12 months was only noted for coffee substitutes (32 % increase). Other studies have shown a slight HMF formation during storage at low temperatures, but a significant increase at 35 °C [21]. For the instant coffee and for coffee substitutes, the average acrylamide concentration was 145 μg/kg and 68.4 μg/kg before storage, respectively (Table 3). During storage at 25 °C for 12 months, AA concentrations in instant coffee and coffee substitutes significantly dropped to 97.0 (33 % reduction) and 49.2 μg/kg (28 % reduction), respectively, indicating the instability of acrylamide in coffee during storage. The same observation was noted in other studies. Delatour et al. [5] reported that a sample of roasted coffee with 203 μg AA/kg, after storage for seven months contained only 147 μg AA/kg. Hoenicke and Gatermann [7] reported AA reduction from 305 μg/kg to 210 μg/kg in roasted coffee for three months of storage at 10–12 °C. A reduction of AA in dried chicory and instant coffee was also found. During storage for several months, acrylamide concentrations dropped significantly, from 214 to 174 μg/kg and 771 to 256 μg/kg, respectively [5].

**Table 3:** Changes of HMF (mg/kg), AA (μg/kg) and water activity (a_w_) in coffee products during storage

Type of product	Storage time (months)	Parameter^1^	Temperature	
			4 °C	25 °C
Instant (soluble) coffee (*n* = 10)	0	HMF AA a_w_	120.0 ± 46.0^Aa^ 145.0 ± 31.0^Aa^ 0.320 ± 0.070^Aa^	120.0 ± 46.0^Aa^ 145.0 ± 31.0^Aa^ 0.320 ± 0.070^Aa^
	6	HMF AA a_w_	128.0 ± 34.0^Aa^ 128.0 ± 26.0^Aa^ 0.319 ± 0.035^Aa^	136.0 ± 32.0^Aa^ 116.0 ± 17.0^Aa^ 0.319 ± 0.033^Aa^
	12	HMF AA a_w_	135.0 ± 24.0^Aa^ 119.0 ± 16.0^Aa^ 0.319 ± 0.028^Aa^	154.0 ± 27.0^Aa^ 97.0 ± 12.0^Bb^ 0.318 ± 0.026^Aa^
Coffee substitutes (*n* = 10)	0	HMF AA a_w_	397.0 ± 71.0^Aa^ 68.4 ± 10.9^Aa^ 0.350 ± 0.100^Aa^	397.0 ± 71.0^Ab^ 68.4 ± 10.9^Aa^ 0.350 ± 0.100^Aa^
	6	HMF AA a_w_	432.0 ± 67.0^Aa^ 63.5 ± 8.5^Aa^ 0.349 ± 0.075^Aa^	456.02 ± 62.0^Ab^ 58.1 ± 7.7^Aa^ 0.347 ± 0.083^Aa^
	12	HMF AA a_w_	469.0 ± 54.0^Ba^ 59.9 ± 8.3^Aa^ 0.348 ± 0.066^Aa^	524.0 ± 51.0^Aa^ 49.2 ± 7.1^Bb^ 0.346 ± 0.069^Aa^

^1^Mean values ± standard deviation (*n* = 10, *n* = the number of the same kind products from different producers)

^A–B^in a row, different letters indicate significant differences (*p* < 0.05)

^a–c^in a column within the same parameter, different letters indicate significant differences (*p* < 0.05) for the same type of products; LSD interaction time–temperature factor of HMF, AA and a_w_ are 1.21, 0.75 1.40 respectively for instant coffee; LSD interaction time–temperature factor of HMF, AA and a_w_ are 0.90, 0.55 1.45 respectively for coffee substitutes

Crisp bread contained 330 mg HMF/kg, on average, while soft bread had 130 mg/kg (Table 4). According to Abraham et al. [18], the HMF content in soft bread can ranged from 14 to 53 mg/kg. The highest HMF content was determined in crisp bread, where the toasting temperature and other production technological parameters differed from those used in traditional bread production and were generally more drastic [18]. The initial content of HMF in crisp bread increased throughout the 12 months of storage with statistically significant changes at high temperature (25 °C) (Table 4). Storage at low temperature (4 °C) did not favour HMF formation throughout the entire studied period. In the case of soft bread with high water activity (0.940), storage at both temperatures (4 and 25 °C) did not favour HMF formation during the 30-day period (Table 4).

**Table 4:** Changes of HMF (mg/kg), AA (μg/kg) and water activity (a_w_) in bread during storage

Type of product	Storage time (months/days^2^)	Parameter^1^	Temperature	
			4 °C	25 °C
Crisp bread (*n* = 10)	0	HMF AA a_w_	330.0 ± 80.0^Aa^ 443.0 ± 110.0^Aa^ 0.282 ± 0.098^Aa^	330.0 ± 80.0^Ab^ 443.0 ± 110.0^Aa^ 0.282 ± 0.098^Aa^
	6	HMF AA a_w_	354.0 ± 41.0^Ba^ 428.0 ± 94.0^Aa^ 0.290 ± 0.095^Aa^	396.0 ± 39.0^Aab^ 374.0 ± 91.0^Aab^ 0.292 ± 0.093^Aa^
	12	HMF AA a_w_	377.0 ± 35.0^Ba^ 416.0 ± 78.0^Aa^ 0.297 ± 0.108^Aa^	434.0 ± 37.0^Aa^ 324.0 ± 70.0^Bb^ 0.299 ± 0.103^Aa^
Soft bread (*n* = 10)	0	HMF AA a_w_	130.0 ± 80.0^Aa^ 55.5 ± 15.5^Aa^ 0.940 ± 0.021^Aa^	130.0 ± 80.0^Aa^ 55.5 ± 15.5^Aa^ 0.940 ± 0.021^Aa^
	15	HMF AA a_w_	134.0 ± 70.0^Aa^ 56.8 ± 14.3^Aa^ 0.938 ± 0.015^Aa^	137.0 ± 58.0^Aa^ 55.3 ± 14.1^Aa^ 0.931 ± 0.023^Aa^
	30	HMF AA a_w_	140.0 ± 61.0^Aa^ 59.2 ± 13.8^Aa^ 0.937 ± 0.018^Aa^	144.0 ± 69.0^Aa^ 53.7 ± 13.1^Aa^ 0.921 ± 0.020^Aa^

^1^Mean values ± standard deviation (*n* = 10, *n* = the number of the same kind products from different producers); ^2^ Storage time of soft bread

^A–B^in a row, different letters indicate significant differences (*p* < 0.05)

^a–c^in a column within the same parameter, different letters indicate significant differences (*p* < 0.05) for the same type of products; LSD interaction time–temperature factor of HMF, AA and a_w_ are 1.47, 0.48, 1.35, respectively for crisp bread; LSD interaction time–temperature factor of HMF, AA and a_w_ are 1.02, 0.85, 1.15, respectively for soft bread

The average acrylamide concentration in the analysed crisp bread was 443 μg/kg before storage while for soft bread it was 55.5 μg/kg (Table 4). Low moisture and large specific surface area of crisp bread contribute to the generally higher acrylamide level in these products. In another study, the content of AA in soft bread and crisp bread was 30–160 and 30–1900 μg/kg, respectively [22]. The highest levels of AA reduction in crisp bread were found in samples stored at 25 °C for 6 months (14 %) and 12 months (27 %), while in the samples stored at 4 °C it was only 3 % and 6 %, respectively (Table 4). In the case of soft bread with high water activity (0.940), a short storage time (15 and 30 days) at both temperatures did not favour AA degradation. The changes in AA content during storage of cereal-based products has been studied by other researchers. They found no common pattern in the degree of AA reduction. The level of AA in some of the cereal-based products was quite stable [5, 6], while others showed a wide range of decrease [3].

Our study showed that acrylamide is not a stable compound in many processed plant products with a long shelf-life. In food products during storage, probably a partial AA degradation takes place and its content decreases. The mechanism of these reactions is not clear. Acrylamide evaporation should be excluded since the products were stored in retailed closed packaging. AA reduction during storage as a result of its polymerization under UV light also has to be excluded because the package of the stored products protected them from light. According to Hoenicke and Gatermann [7], acrylamide reactions with compounds containing SH groups, which are particularly present in food products such as coffee and cocoa or milk powder, can have a significant impact on AA reduction during storage in these products or in foods with their addition. Storage affects AA degradation in coffee (45–60 %), cacao (32 %) and foods fortified with milk powder (29 %). Generally, it is believed that the high reactivity of acrylamide toward nucleophilic food components, such as sulfhydryl, amino and hydroxyl groups of peptides, proteins, and melanoidins in acrylamide-containing foods, might be responsible for the reduction of AA in stored food products [7]. Consequently, acrylamide could be quite stable in food such as cereal-based products (bread, breakfast cereals and biscuits), that usually do not consist of sulphur-containing substances [5, 6]. On the other hand, some authors have reported that storage temperature affects AA degradation in the crisp bread (37 %) and baby and dietary biscuits (7 and 11 %, respectively) [3, 7]. They suggested that water content could be the major factor responsible for AA reduction during storage. It is believed that the varying results of AA found in stored food products of some researchers depend not only on differences in food composition, but also on the water content in these products. It seems that processes for acrylamide elimination are more complicated.

The results of this study showed that the AA content in the examined products was stable during storage at low temperature. A higher storage temperature, however, resulted in a reduction of AA content. Statistical analysis revealed that the interaction time temperature factor had a very strong effect (*p* < 0.05) on AA content for most of the stored products. Moreover, it was also possible to conclude that a reduction in AA content was influenced by moisture content and the kind of product. The initial low water activity of the products resulted in a higher decrease in the acrylamide content during storage. A negative correlation (−0.66) was noted between AA and initial a_w_ (Table 5). This study also confirmed that the product composition (sulphur-containing substances) may have a significant impact on AA reduction in stored food. ɛ-NH_2_ and SH groups of amino acids present in products might be responsible for the high degradation of acrylamide in 100 cocoa and 20 % cocoa (51 and 35 % decrease, respectively), instant coffee and coffee substitutes (33 and 28 % decrease, respectively) and instant baby foods which contained milk powder (39 % decrease) (Tables 2, 3 and 1, respectively). These examined products contain a large quantity of sulphur amino acids, whose reactions with acrylamide can be assumed as the cause of the significant AA degradation during storage. In addition, it seems that, just like in cocoa products (100 % and 20 % cocoa), a higher content of special substances in instant coffee than in coffee substitutes might be responsible for a greater acrylamide reduction in coffee-rich products. These observations are important as they may provide an avenue for studies to identify certain constituents in the food matrixes that may potentially “inactivate” or eliminate acrylamide. In addition, it can be concluded that the often-noted differences in acrylamide content in the same type of products may result from various times and storage temperatures prior to analysis, different initial moisture levels and also from differences in the product composition.

**Table 5:** Correlation matrix between HMF, AA and initial water activity (a_w_)

	HMF	AA	a_w_
HMF	1.00		
AA	−0.74^*^	1.00	
a_w_	−0.36	−0.66^*^	1.00

^*^Correlation coefficients statistically significant at *p* < 0.05

Our study also showed that prolonged storage enhanced HMF formation in some processed plant products with long shelf-life. A large increase in HMF during storage was observed at a higher temperature, which indicates a large dependence of HMF formation on storage time and temperature. Statistical analysis revealed that the interaction time–temperature factor had a significant effect (*p* < 0.05) on HMF content. In addition, it was found that an a_w_ of about 0.4 is favourable to HMF formation. The highest level of HMF formation was found in storage products with an initial water activity in the range of 0.3–0.4. There was a statistically negative correlation (*p* < 0.05) between HMF and initial a_w_ (Table 5). It was noted that with very high and low product water activity, HMF formation was limited during the whole study period. Hence, this result confirms the significance of water activity in HMF formation. Water may act partly as a reactant and partly as a solvent and transporting medium for reactants (reactant mobility). At very high and low water activity values, the reactivity of reactants is limited, probably due to high dilution and their low mobility (despite the presence at high concentrations), respectively. The differences in HMF increase observed in the analysed samples after storage may also be due to differences in the product compositions. As is well-known, HMF content in food during storage may be the result of complex reactions leading to both formation and degradation of this molecule. The mechanism of such reactions is fairly complex and may be different in various products. A product moment correlation test between HMF and AA content showed a strong negative correlation *i.e.*, -0.74 (Table 5). The results of this work clearly demonstrate that those two parameters (AA and HMF level) are directly related to product storage and are inversely proportional to each other during storage.

## Conclusions

Acrylamide content decreases in some foods during long storage, particularly at higher temperatures. Further studies are needed to investigate the mechanism of acrylamide reduction during warm storage and the possibility of applying this procedure to appropriate products. The challenge is to find a balanced approach between acrylamide reduction and HMF formations in products during storage.

## Abbreviations

AA: Acrylamide
ANOVA: Analysis of variance
a_w_: Water activity
DAD: Diode array detector
HMF: 5-hydroxymethylfurfural
LSD: Least significant difference test
RP-HPLC: Reversed phase-high performance liquid Chromatography
SD_s_: Standard deviations
